# Mean Fetal Kidney Length at the Third Trimester: An Emerging Ultrasound Parameter for Gestational Age Assessment

**DOI:** 10.7759/cureus.77796

**Published:** 2025-01-21

**Authors:** Sasikala Kathiresan, Susithira Aarthy, Emil Phinehas, Kalaiselvi Selvaraj

**Affiliations:** 1 Department of Obstetrics and Gynecology, All India Institute of Medical Sciences, Madurai, Madurai, IND; 2 Department of Obstetrics and Gynecology, Susi Hospital, Sivagiri, IND; 3 Department of General Surgery, All India Institute of Medical Sciences, Madurai, Madurai, IND; 4 Department of Community and Family Medicine, All India Institute of Medical Sciences, Madurai, Madurai, IND

**Keywords:** biometric parameter, fetal kidney length, gestational age, third trimester, ultrasound

## Abstract

Introduction

Accurate gestational age (GA) determination is essential for effective obstetric care, guiding the timing of delivery, fetal evaluations, and interventions. Conventional ultrasound (USG) markers such as biparietal diameter (BPD), head circumference (HC), abdominal circumference (AC), and femur length (FL) often lose precision in the third trimester, with discrepancies of up to three weeks. These limitations highlight the need for alternative, reliable metrics. Fetal kidney length (FKL), which increases consistently by about 1 mm per week after 24 weeks of gestation and remains unaffected by growth restrictions, presents a promising alternative. This study aimed to evaluate the diagnostic utility of mean FKL for third-trimester GA assessment.

Methods

A cross-sectional study was conducted from March to June 2021 at a tertiary care hospital in Tamil Nadu, India. Fifty pregnant women with singleton pregnancies between 28 and 42 weeks of gestation were enrolled. Participants were required to have confirmed last menstrual period (LMP) dates and first-trimester dating scans. Pregnancies with high-risk factors, fetal anomalies, or other complications were excluded. Biometric parameters (BPD, HC, AC, and FL) and mean FKL were measured using USG. FKL was obtained by averaging the lengths of both kidneys in the paravertebral plane. FKL-based GA (FKLGA) was compared with LMP-based GA (LMPGA) and GA derived from conventional markers. Analyses included intraclass correlation coefficients (ICC), linear regression, and Bland-Altman plots.

Results

The mean age of participants was 23.6 ± 1.6 years, and 54% were primigravidae. The median LMPGA was 37 weeks (interquartile range (IQR): 35-39 weeks), closely aligned with FKLGA. The mean FKL was 36.3 ± 3.2 mm. Bland-Altman plots showed that FKLGA had narrower limits of agreement (LoA) (-0.5 to 1 week) compared to conventional parameters (-2 to 6 weeks). The ICC between FKLGA and LMPGA was 0.986 (95% confidence interval (CI): 0.976-0.992), significantly higher than that of conventional markers (0.539; 95% CI: 0.31-0.739). Linear regression showed that FKL explained 97.4% of GA variability (adjusted R² = 0.974), with a 1 mm increase in FKL corresponding to a one-week GA increase (β = 0.99; p < 0.001). Conventional markers explained only 57.3% of GA variability (adjusted R² = 0.573).

Discussion

This study underscores the reliability of FKL for third-trimester GA estimation. The strong correlation between FKLGA and LMPGA, demonstrated by high ICC and narrow limits of agreement, supports its clinical utility. Unlike conventional biometric parameters, FKL remains unaffected by growth restrictions, making it particularly valuable for late-presentation pregnancies or uncertain LMP dates. Incorporating FKL into USG protocols can address the limitations of conventional markers and improve decision-making in high-risk pregnancies.

Conclusion

Mean FKL is a reliable and reproducible parameter for estimating GA in the third trimester. It outperforms conventional markers and closely aligns with LMPGA, offering a robust alternative for late-pregnancy evaluations. Further research should validate its application across diverse populations and integrate it into predictive models for enhanced clinical accuracy.

## Introduction

Accuracy in the calculation of gestational age (GA) is the cornerstone in obstetric care that drives the treatment plan in terms of time and mode of delivery. It also provides the critical window for various screening tests and assessment of the fetus in the continuum of care during pregnancy. Inappropriate GA assessment results in either underestimation of potential risks in fetuses such as intrauterine growth restriction (IUGR) or poorly timed interventions such as induction of labor or cesarean section. Women who present late in pregnancy with uncertain or unknown menstrual dates rely on ultrasound (USG) for GA, which is the least reliable after the first trimester. Hence, careful consideration of the entire clinical picture is required in management decisions when based on third-trimester ultrasound alone.

In practice, gestational assessment with the last menstrual period (LMP) along with a first-trimester dating scan by measuring crown-rump length ascertain GA accurately [1]. However, GA in the second and third trimesters assessed by conventional biometric parameters (CBP) in ultrasound, such as head circumference (HC), biparietal diameter (BPD), abdominal circumference (AC), and femur length (FL), is with disparities. This can be as high as three weeks in the third trimester [2]. As a result, increasing GA is an independent risk factor for inappropriate GA assessment by CBP in ultrasonography (USG) [3]. Thus, there is a need for more accurate estimates of gestational age through better diagnostic parameters.

Fetal kidney development has demonstrated a consistent increase of 1.7 mm every two weeks during pregnancy, remaining unaffected by any growth abnormalities [4]. As reported by Konje et al., fetal kidney length (FKL) correlates positively with gestational age after 24 weeks [5]. Further, FKL is an easily reproducible parameter in third-trimester ultrasound [6]. Unlike other conventional biometric parameters, FKL does not vary with fetal growth restriction compared to appropriately grown fetuses [7]. In this context, this study aimed to ascertain the diagnostic utility of mean FKL measurement in the determination of GA in the third trimester.

Hence, the aims and objectives of this study are as follows: to estimate the intraclass correlation between mean fetal kidney length (FKL) measurement in the determination of gestational age (GA) using ultrasonography (USG), to assess the reliability of mean FKL measurement in the determination of gestational age against other conventional biometric parameters (BPD, HC, AC, and FL) by ultrasonography, and to build the model to predict gestational age using mean FKL measurement.

## Materials and methods

Study design and setting

This was a facility-based cross-sectional study conducted in a tertiary care hospital in Madurai, Tamil Nadu. This facility caters to two lakh reproductive population approximately. The annual footfall of antenatal women registered and receiving antenatal care from this facility is around five hundred. Trained obstetricians and radiologists perform the ultrasound in this facility.

Study population and sampling method

After obtaining Institutional Ethics Committee (IEC) approval (approval number: VMCIEC/01/2021, date: 24/02/2021), this study included antenatal pregnant women who had 28 completed weeks but within 42 weeks of the gestation period, were not classified under any high-risk pregnancy categories, had singleton pregnancy, and were able to accurately recall the last menstrual period (LMP) with corresponding gestation confirmed in the first-trimester dating scan done between eight and 12 weeks of gestation. Pregnant women with an anomalous fetus, suspected IUGR, unknown dates, multiple gestations, gestational diabetes mellitus, oligo-/polyhydramnios, fetal hydroureteronephrosis and other renal abnormalities, and failure to visualize biometric parameters were excluded from the study. All pregnant women referred for antenatal USG in the facility, during the reference period from March to June 2021, were consequently enrolled without further sampling. A total of 50 third-trimester antenatal women were included in the study after applying the inclusion and exclusion criteria.

Data collection

One trained operator (radiologist with five years experience) performed all examinations using a 2-5 MHz curvilinear probe in a Philips HD5 ultrasound machine (Bothell, WA). The conventional biometric parameters (BPD, HC, AC, and FL) were measured in the USG, based on the standard methods described in Callen's Ultrasonography in Obstetrics and Gynecology [8]. Mean FKL refers to the whole length of both kidneys separately in the paravertebral plane; the values were averaged. All parameters were taken during restricted/minimal fetal movements. Data were collected in a structured data extraction sheet. The variables included were the age of the pregnant woman, LMP, GA based on LMP, conventional biometric parameters as per USG (BPD, HC, AC, and FL), GA as per conventional parameters, mean FKL, and GA as per FKL.

Data management

Data were entered in an Excel spreadsheet (Microsoft Corp., Redmond, WA). Clinical and demographic features were summarized as frequencies and percentages. Biometric parameters were described as mean and standard deviation (SD). Using the linear regression model, the equation for estimating gestational age from mean FKL was obtained. In the linear regression, the actual gestational age was the dependent variable and FKL was the independent variable. The Bland-Altman plot was drawn between predicted gestational age using mean FKL and actual gestational age based on LMP with limits of agreement (LoA) including difference ±2 standard error limits. Similarly, gestational age based on other fetal parameters and LMP-based gestational age were also plotted. Intraclass correlation measures were estimated. All analyses were done using Stata software (StataCorp LLC, College Station, TX), and plots were created in R software.

## Results

A total of 50 antenatal women were enrolled in the study for the assessment of GA using various ultrasonogram-based parameters. About 54% of them were primigravidae, and 8% of the study population had a previous history of abortion. The mean (SD) age of the participants was 23.6 (1.6) years. Other obstetric parameters of the participants are described in Table 1, and the distribution of biometric parameters in ultrasonogram is listed in Table 2.

**Table 1:** Distribution of characteristics of antenatal women who participated in the study (N = 50) SD: standard deviation

Factor	Frequency (number)	Percentage (%)
Gravida		
Primigravida	27	54
2	10	20
3 or more	13	26
Para		
Nullipara	27	54
1	13	26
2 or more	10	20
Previous history of abortion status		
No	46	92
Yes	4	8
Age mean (SD)	23.6 (1.6)	

**Table 2:** Distribution of biometric parameters observed in USG (N = 50) USG: ultrasound, BPD: biparietal diameter, AC: abdominal circumference, HC: head circumference, FL: femur length, FKL: fetal kidney length, SD: standard deviation

Parameter	Mean	SD	P25	P75
BPD (cm)	8.57	0.52	8.25	8.9
AC (cm)	31.3	2.3	30.2	32.4
HC (cm)	31.4	1.6	30	32.5
FL (cm)	6.6	0.6	6.2	7.1
Mean FKL (mm)	36.3	3.2	33.7	38.6

**Figure 1:**
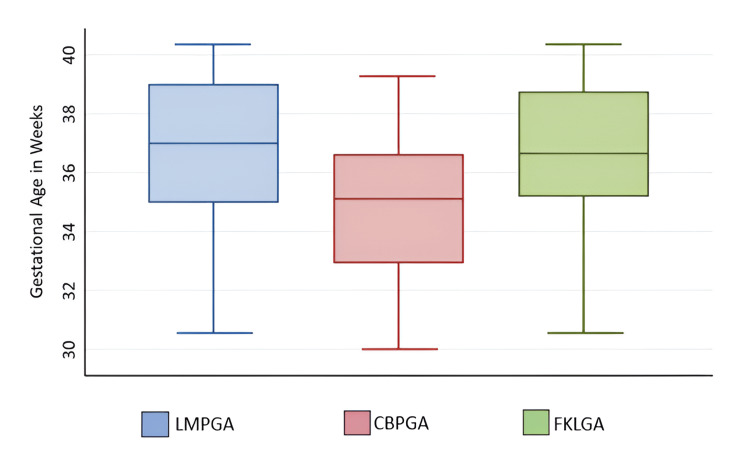
Distribution of gestational age based on different parameters Each box represents the IQR, which contains the middle 50% of the data (from the 25th to the 75th percentile), whereas the line inside the box indicates the median gestational age for each method. LMPGA has the highest median gestational age, and CBPGA has the lowest median, indicating that it may systematically underestimate gestational age compared to FKLGA. LMPGA: last menstrual period-based gestational age, CBPGA: conventional biometric parameter-based gestational age, FKLGA: fetal kidney length-based gestational age, IQR: interquartile range

The median gestational age based on LMP (LMPGA) was 37 weeks with an interquartile range (IQR) from 35 to 39 weeks. The IQR of mean fetal kidney length-based gestational age (FKLGA) varied from 35 to 38 weeks. The average gestational age based on conventional biometric parameters (CBPGA) (BPD, HC, AC, and FL) was lower compared to LMPGA or FKLGA (Figure 1).

**Figure 2:**
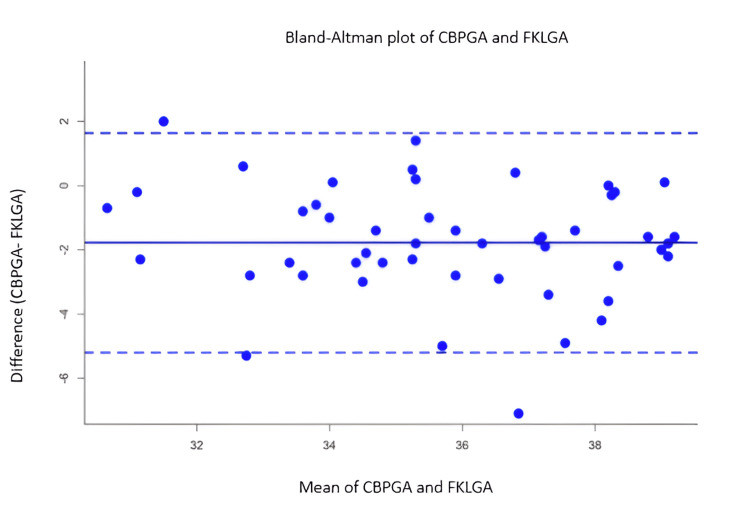
Bland-Altman plot showing the mean and the difference between CBPGA and FKLGA The mean of CBPGA and FKLGA in the X-axis represents the average gestational age estimated by the two methods, whereas the difference between CBPGA and FKLGA in the Y-axis shows the difference in gestational age estimates between CBPGA and FKLGA for each data point. The mean difference is slightly negative, suggesting that, on average, FKLGA estimates are slightly higher than CBPGA. Most of the data points lie within the limits of agreement (±1.96 SD), which suggests that the two methods generally agree. CBPGA: conventional biometric parameter-based gestational age, FKLGA: fetal kidney length-based gestational age, SD: standard deviation

**Figure 3:**
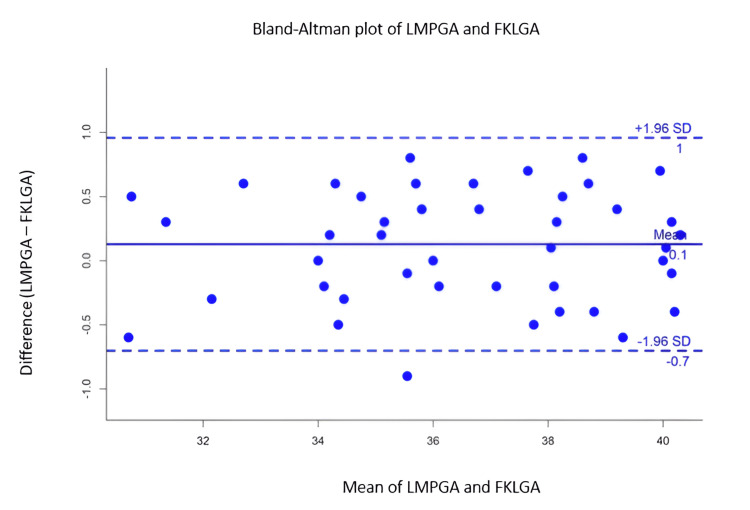
Bland-Altman plot showing the mean and the difference between LMPGA and FKLGA The mean difference is close to zero (+0.1), indicating almost no systematic bias between LMPGA and FKLGA, suggesting that FKLGA provides gestational age estimates that align closely with LMPGA on average. The small mean difference and narrow limits of agreement indicate that LMPGA and FKLGA are comparable methods for estimating gestational age. LMPGA: last menstrual period-based gestational age, FKLGA: fetal kidney length-based gestational age

In Figure 2 and Figure 3, the Bland-Altman plot was drawn between the average of reference gestational age (LMPGA) and index markers such as FKL and conventional biometric parameters in the X-axis and the difference between reference (LMPGA) and conventional biometric parameters in the Y-axis. The limits of agreement (LoA) fall between -0.5 and 1 week in the mean FKL-based index markers, whereas the LoA varied from -2 to 6 weeks in the other markers based on gestational age.

**Figure 4:**
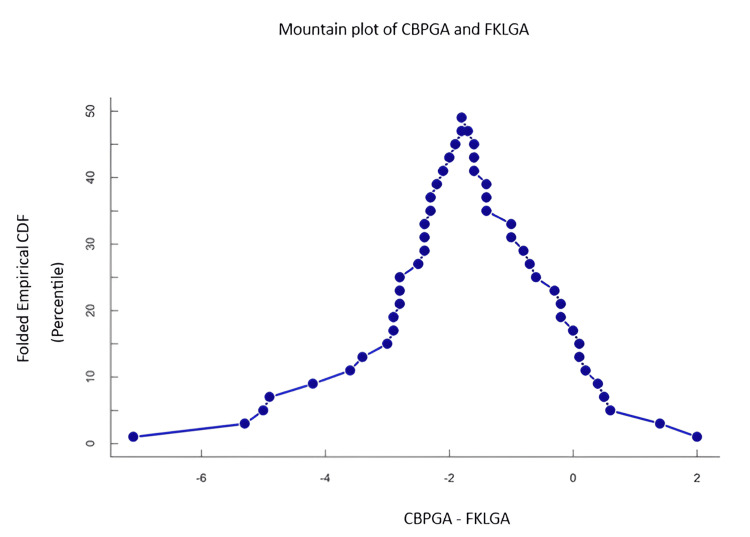
Mountain plot showing the difference between CBPGA and FKLGA The X-axis represents the difference in gestational age estimates between CBPGA and FKLGA, whereas the Y-axis represents the CDF in a folded or symmetric manner around the peak. The central tendency is near -2, indicating that the most frequent difference between CBPGA and FKLGA is approximately -2 weeks, suggesting that, on average, CBPGA underestimates gestational age compared to FKLGA. A narrow and steep curve suggests better agreement, as the differences are clustered around the peak value. CBPGA: conventional biometric parameter-based gestational age, FKLGA: fetal kidney length-based gestational age, CDF: cumulative distribution function

**Figure 5:**
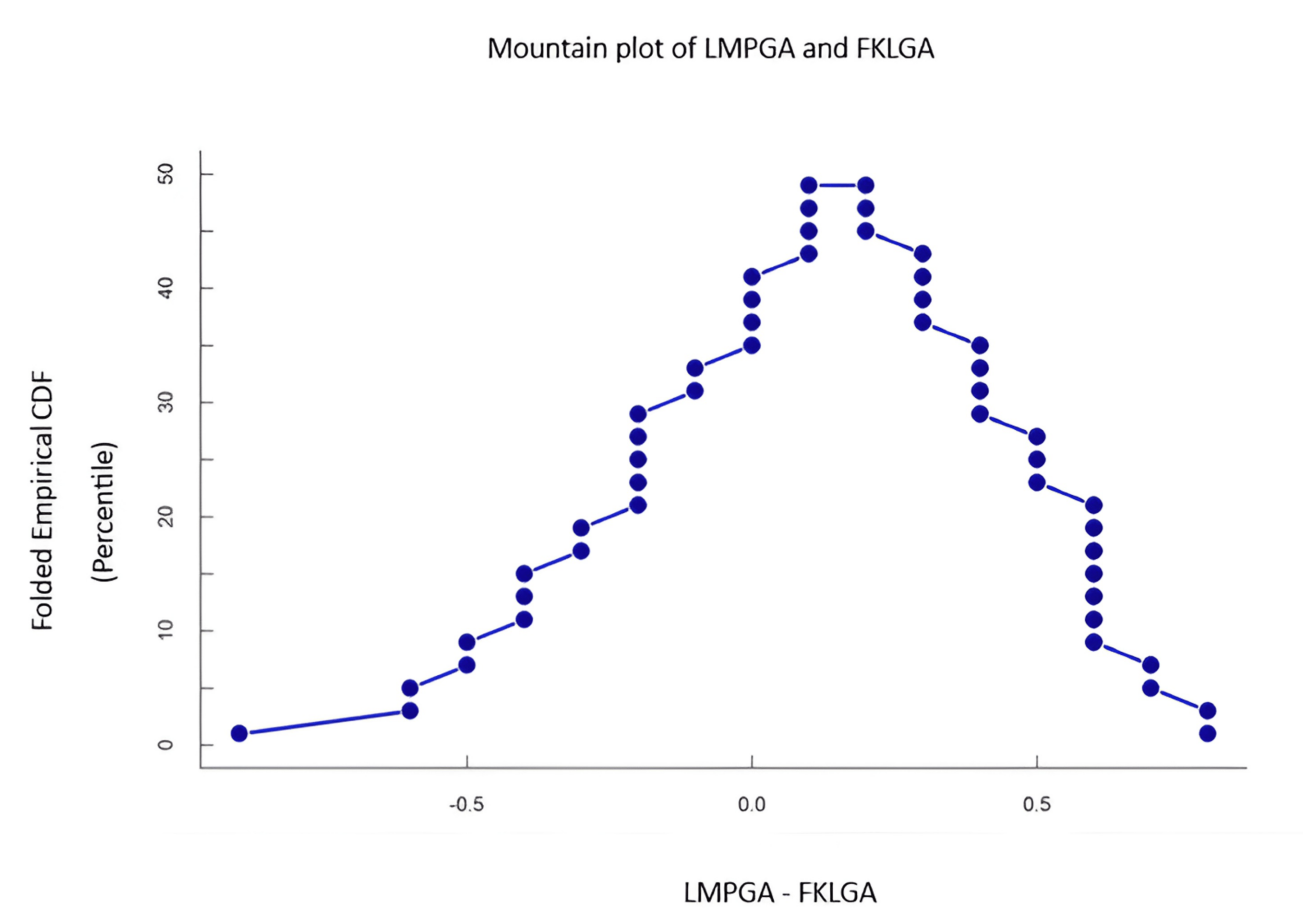
Mountain plot showing the difference between LMPGA and FKLGA The central tendency is around 0, indicating that the most frequent difference between LMPGA and FKLGA is approximately 0 weeks, suggesting a good overall agreement between the two methods. The narrow and steep curve indicates strong agreement between the methods, with minimal variability. LMPGA: last menstrual period-based gestational age, FKLGA: fetal kidney length-based gestational age, CDF: cumulative distribution function

In Figure 4 and Figure 5, the mountain plot based on difference and cumulative distribution function also showed the peak around 0 in mean FKLGA, whereas the peak was observed at 2 weeks in other markers. The consistent difference (centered near -2 weeks) indicates that CBPGA systematically underestimates gestational age compared to FKLGA.

The intraclass correlation between LMPGA and mean FKLGA was 0.986 (95% CI: 0.976-0.992). The intraclass correlation between LMPGA and GA based on other markers was 0.539 (95% CI: 0.31-0.739). The linear regression model was built considering LMPGA as a dependent variable and maternal age and index markers (mean FKLGA and GA based on other markers) as independent variables. Maternal age was found to be insignificant. The fetal kidney-based model explained around 97.4% variability in the gestational age (adjusted squared R - 0.9738). Each one-unit increase in fetal kidney length correspondingly increased one week of gestational age (beta coefficient: 0.99; 95% CI: 0.94-1.04; p = 0.001). In the linear regression, gestational age based on other markers explained 57.3% of the variability in the gestational age (beta coefficient: 0.85; 95% CI: 0.64-1.06; p = 0.001; adjusted squared R - 0.5729).

## Discussion

The current study carried out among third-trimester pregnant women showed the following key features: (1) the high intraclass correlation observed between mean FKLGA and LMPGA compared to other traditional USG-based fetal biomarkers; (2) about 97% of the variability in gestational age was explained by fetal kidney length alone, whereas other markers, such as BPD, HC, AC, and FL, were able to explain around 57%; and (3) a one-unit (1 mm) increase in FKL corresponds to one week of maturity in GA [9].

Gestational age is the single most predominant factor that determines the course of action in managing high-risk pregnancies and determines the prognosis. The favorable maternal and fetal outcomes are dependent on these key decisions. However, in the context where not all registration of pregnancies are within the first trimester and often proportion of antenatal women recalling their LMP is less than 80% [3], the reliance on USG-based fetal markers cannot be overlooked.

Although USG plays a vital role in GA assessment, its accuracy is limited to the first trimester. For pregnant women presenting late or uncertain LMP dates, third-trimester conventional biometric parameters are less reliable in GA assessment. Rumack and Levine noted in their textbook that the kidneys continue to develop during pregnancy [10]. They provide a nomogram illustrating renal lengths from 14 to 42 weeks of gestation, indicating that the renal-to-abdominal circumference ratio remains stable at 0.27-0.30 throughout pregnancy. The mean FKL in assessing GA is in close correlation with GA by LMP in comparison with GA assessment with other conventional biometric parameters in USG [11]. Kidneys are identified easily and measured in late pregnancy.

The gestational age in the third trimester was estimated using LMP, CBP, and mean FKL in this study. It is observed that LMPGA might tend to overestimate gestational age, while CBPGA might underestimate it. FKLGA lies in between, potentially making it a balanced option. This study shows that the length of the mean fetal kidney length and gestational age linearly increases from 29.5 ± 3 mm at 31 weeks to 40 ± 3 mm at 40 weeks, which is similar to the study conducted by Peter et al. in the Indian population [12]. The current study reports a high ICC between LMPGA and FKLGA, which was 0.986, whereas CBPGA has an ICC of 0.539. These measurements are close to the findings of Yusuf et al. [13] and Bardhan et al. [14]. Evidence from various other parts of the world has reported between 20 and 40 weeks of FKL, which acts as a reliable marker [15]. The high level of agreement implies that both LMPGA and FKLGA can be used in most clinical scenarios for GA assessment; however, given the small differences, FKLGA can be considered a practical alternative to LMPGA, especially in scenarios where LMP data are unavailable or uncertain. The rate of increase in FKL reported in the current study (1 mm increase per GA week maturity) corresponds to the coefficient level reported by Kansaria and Parulekar (0.9 mm/GA week) [4]. This is consistent with the study by Cohen et al. that kidney length correlates with gestation [16,17]. Konje et al. predicted the gestational age close to ±8 days in the third trimester by combining FKL and conventional biometric parameters [5].

The study included only 50 participants from a single center, which may limit the generalizability of the findings. Larger, more diverse populations are necessary to validate the results. High-risk pregnancies were excluded, which may limit the applicability of the findings to more complex clinical cases where accurate GA estimation is often more critical.

## Conclusions

This research highlights the effectiveness of mean FKL as an ultrasonographic measure for accurately determining GA during the third trimester. With an impressive intraclass correlation of 0.986 between FKLGA and LMPGA, FKL surpasses traditional biometric indicators such as biparietal diameter, head circumference, abdominal circumference, and femur length, which tend to exhibit higher variability and lower reliability. The consistent linear growth pattern of FKL aligns closely with GA, making it an essential tool for pregnancies that present late or have uncertain LMP dates. The findings indicate that FKL accounts for 97% of the variability in GA, providing a crucial advantage in clinical scenarios where precise GA estimation is vital for timely and appropriate obstetric interventions. Further studies with diverse populations are needed to replicate the analysis across different subgroups to confirm the generalizability of this agreement. Integrating FKL into standard third-trimester ultrasonographic evaluations could enhance maternal and fetal outcomes by delivering more accurate GA measurements, particularly when other methods are inadequate.
